# An efficient, flexible perovskite solar module exceeding 8% prepared with an ultrafast PbI_2_ deposition rate

**DOI:** 10.1038/s41598-017-18970-y

**Published:** 2018-01-11

**Authors:** Kunpeng Li, Junyan Xiao, Xinxin Yu, Tianhui Li, Da Xiao, Jiang He, Peng Zhou, Yangwen Zhang, Wangnan Li, Zhiliang Ku, Jie Zhong, Fuzhi Huang, Yong Peng, Yibing Cheng

**Affiliations:** 1 State key lab of advanced technology for materials synthesis and processing, Wuhan University of Technology, Wuhan, 430070 China; 2 Hubei key laboratory of low dimensional optoelectronic materials and devices, Hubei University of Arts and Science, Xiangyang, 441053 China; 3 Department of materials science, Monash University, Melbourne, VIC 3800 Australia

## Abstract

Large-area, pinhole-free CH_3_NH_3_PbI_3_ perovskite thin films were successfully fabricated on 5 cm × 5 cm flexible indium tin oxide coated polyethylene naphthalate (ITO-PEN) substrates through a sequential evaporation/spin-coating deposition method in this research. The influence of the rate-controlled evaporation of PbI_2_ films on the quality of the perovskite layer and the final performance of the planar-structured perovskite solar cells were investigated. An ultrafast evaporation rate of 20 Å s^−1^ was found to be most beneficial for the conversion of PbI_2_ to CH_3_NH_3_PbI_3_ perovskite. Based on this high-quality CH_3_NH_3_PbI_3_ film, a resultant flexible perovskite solar sub-module (active area of 16 cm^2^) with a power conversion efficiency of more than 8% and a 1.2 cm^2^ flexible perovskite solar cell with a power conversion efficiency of 12.7% were obtained.

## Introduction

Flexible, lightweight photovoltaic devices can be employed in numerous emerging areas, such as bendable displays, conformable sensors, biodegradable electronic devices, portable electronic chargers and wearable electronic textiles, thus attracting considerable attention from both research institutes and industries^1^. Among all the traditional and new-generation photovoltaic technologies, perovskite solar cells containing metal halide perovskite materials as an absorber have exhibited advantages that include high efficiency, low cost and low-temperature fabrication, which can guarantee the compatibility with most flexible substrates and practical applications^2–4^.

Currently, the certified power conversion efficiency (PCE) records of perovskite solar cells on rigid substrate can achieve over 22% with an area of 0.1 cm^2^, 19.7% with an area of 1 cm^2^ and 12.1% with an area of 36.1 cm^2^ (module), respectively^5,6^. Flexible perovskite solar cells (F-PSCs) can also reach PCE over 18% in an area of 0.1 cm^2^ ^7^. However, most studies on F-PSCs are based on a typical size of approximately only 0.1 cm^2^. The PCEs of large-area flexible perovskite solar cells, especially flexible perovskite solar modules, still lag behind those of small-area devices^8–10^. Therefore, fabricating an efficient F-PSC with a reasonable size, for example, not less than 5 cm × 5 cm, is essential for enabling PSCs to become commercially available. In all steps of F-PSC module/sub-module fabrication, preparing pinhole-free, uniform perovskite films with large areas and high reproducibility is the most important challenge. This is because either the widely used metal halide predecessor PbI_2_ or the resultant perovskite materials, such as CH_3_NH_3_PbI_3_ (MAPbI_3_), demonstrate specific solution crystallization processes in which the nucleation rates do not match the crystallization rates^11^. Various modified spin-coating techniques, such as “gas-assisted nucleation”^12^, “solvent engineering”^13^, and “vacuum flashing”^14^ methods have been invented to form homogenous perovskite films directly from the perovskite precursor solution for application in small-area devices. However, the possibility to scale up these methods remains questionable because of the complexity of processing. In the case of methods involving the vapor phase, Liu *et al*. demonstrated that a homogeneous perovskite film could be obtained from dual-source evaporation, giving a high PCE of 15.4% in the corresponding device^15^. Unfortunately, this promising approach faced problems of a high vapor pressure and easy decomposition of the organic salt caused by the control of CH_3_NH_3_I (MAI). Fu *et al*. employed a hybrid sequential deposition method with evaporated PbI_2_ and a CH_3_NH_3_I solution^16^, yielding a semi-transparent solar cell with a steady-state efficiency of 14.2% along with a 72% average transmittance in the near-infrared region, which ensures that this strategy has a good prospect.

Herein, a hybrid sequential deposition method was adopted to fabricate module-sized perovskite films on flexible substrates. The effects of the PbI_2_ deposition rates on the quality of the perovskite films and device performance were investigated. At an ultrafast rate of 20 Å s^−1^, large-area flexible perovskite solar cells with an active area of 1.2 cm^2^ were prepared that achieved an efficiency of 12.7%. In addition, a flexible perovskite solar module with an active area of 16 cm^2^ on a 25 cm^2^ ITO-PEN substrate exhibited a PCE of more than 8%. Combining the merits of the low cost, short processing time, full coverage of the substrate, and high module performance, the PbI_2_ film fabricated via ultrafast thermal evaporation holds promise for facilitating the development of industrial-scale perovskite solar cells.

## Results

In this research, fullerene C_60_ was selected as an electron transport material (ETL) and first evaporated onto a indium tin oxide-coated polyethylene naphthalate (ITO-PEN) flexible substrate^17^. Then, PbI_2_ was evaporated on this amorphous C_60_ layer at various controlled deposition rates within the range of 0.5 Å s^−1^ to 40 Å s^−1^, which could be easily adjusted by controlling the evaporation source temperature. Using this thermal evaporation method, a uniform PbI_2_ layer with a precisely controllable amount over a large area could be deposited.

**Figure 1:**
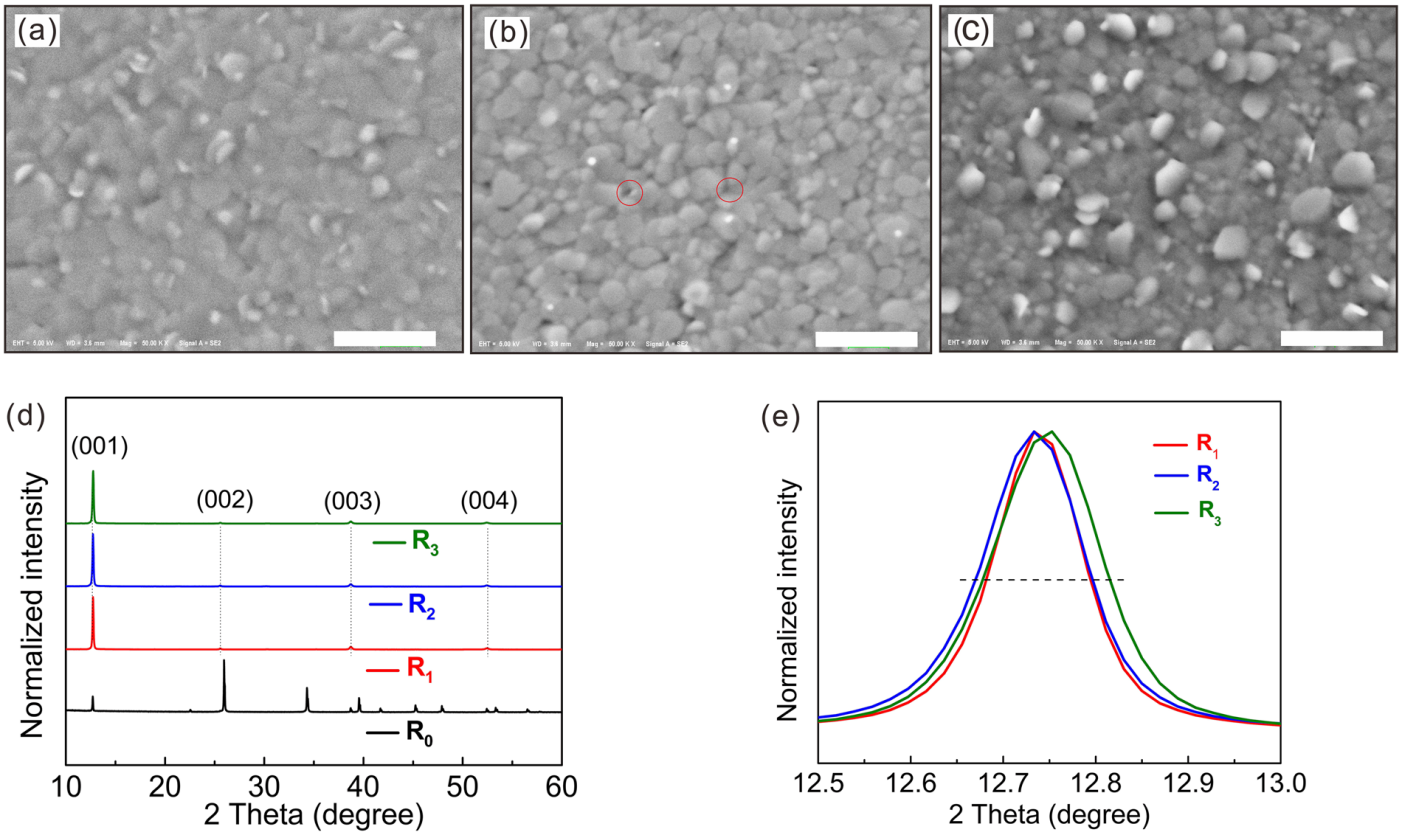
Surface SEM images of the PbI_2_ films thermally deposited at rates of (**a**) R_1_, (**b**) R_2_ and (**c**) R_3_. The scale bars indicate 500 nm. (**d**) Corresponding PbI_2_ XRD patterns and (**e**) magnified XRD patterns between 12.5° and 13°.

Under the naked eyes, all these evaporated PbI_2_ films exhibit a uniform color and an ultrasmooth surface (see Fig. S1 in the supplementary information). However, under scanning electron microscopy (SEM), the microscopic morphologies of the PbI_2_ films vary slightly for the different evaporation rates. It has been reported that the structural properties of evaporated PbI_2_ thin films can be adjusted by varying the deposition conditions, such as the substrate materials, substrate temperature and evaporation rates^18–20^. To impart more significant differences in the PbI_2_ films and in the subsequent perovskite films and devices, the deposition rates were fixed at 0.5 Å s^−1^ (R_1_), 20 Å s^−1^ (R_2_) and 40 Å s^−1^ (R_3_) for detailed investigation. Figure 1(a–c) shows the surface morphologies of the thermally evaporated PbI_2_ films at deposition rates of R_1_, R_2_ and R_3_. The PbI_2_ film prepared with a deposition rate of 0.5 Å s^−1^ is smooth, compact and nearly free of pinholes, as shown in Fig. 1(a). In addition, at the rate of 20 Å s^−1^, the PbI_2_ film is still smooth but less compact. A porous surface and some pinholes can be seen in Fig. 1(b). When the deposition rate increased to 40 Å s^−1^, the film became rough, and many PbI_2_ nanoplates formed on the top surface, as shown in Fig. 1(c), which is similar to the morphology of the crystallized substrate^15^. These nanoplates are randomly arranged perpendicular to the substrate, which can create a large degree of porosity.

X-ray diffraction measurement (XRD) was employed to further investigate the microstructure of the PbI_2_ films. As shown in Fig. 1(d), all the evaporated PbI_2_ layers demonstrate a preferential (001) orientation compared with the raw PbI_2_ powder (R_0_). In the magnified XRD patterns, as the deposition rate rising up, the full width at half maximum (FWHM) of the peak increases slightly. To give a quantitative analysis, the average crystalline sizes of the PbI_2_ films were calculated by the Scherer equation using the (001) diffraction peak through the formula reported elsewhere and shown in Supplementary Table S1. Clearly, these calculated values of crystalline sizes are smaller than the submicron-scaled grain sizes observed from the SEM images^19^. The PbI_2_ film deposited at a rate of R_1_ has the smallest (001) FWHM, meaning it has the largest crystalline size, which forms the most compact layer in Fig. 1(a). Compared with the R_1_-based sample, the R_2_-based sample has more pores in the thin film. However, upon further increasing the deposition rate to R_3_, a rough surface is formed. In brief, by varying the deposition rate, the morphology and microstructure of the PbI_2_ film can be controlled in this work. Moreover, higher deposition rates correspond to shorter deposition times, which is good for manufacturing.

**Figure 2:**
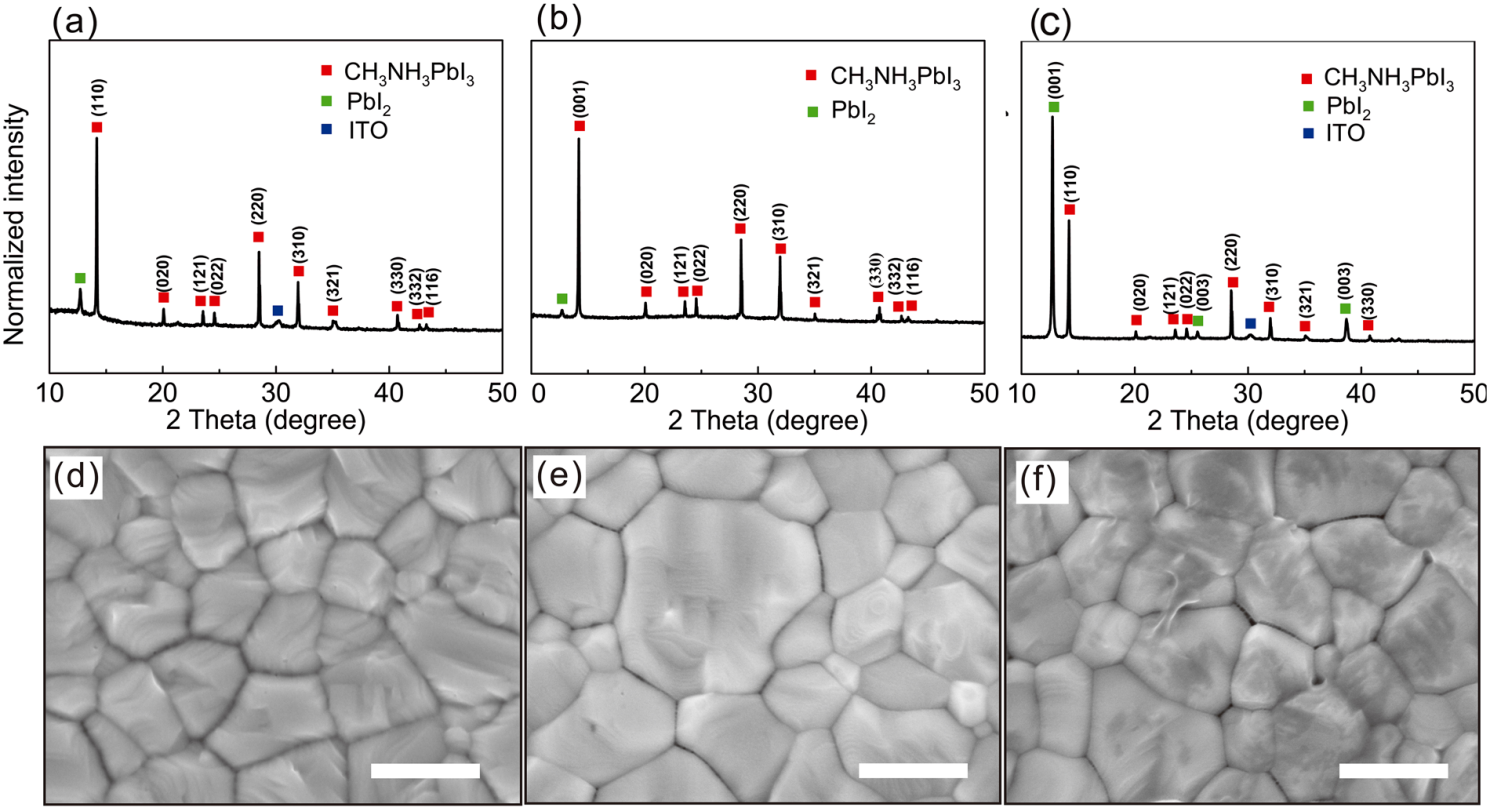
XRD patterns and corresponding surface SEM images of the CH_3_NH_3_PbI_3_ films fabricated from different deposition rates of PbI_2_: (**a** and **d**) R_1_, (**b** and **e**) R_2_ and (**c** and **f**) R_3_. The scale bars indicate 500 nm.

To understand the transformation from PbI_2_ to MAPbI_3_ perovskite, the deposition-rate-dependent PbI_2_ films with different porosities were then transformed to perovskite films by spin-coating the MAI solution and annealing^21,22^. It has been demonstrated that perovskite films fabricated on an amorphous substrate, such as the [6,6]-phenyl-C61-butyric acid methyl ester (PCBM) layer, obtained by here described always contain residual PbI_2_^16,23^. However, the performance of the final device can still be efficient if the amount of residue is controlled^14^. Figure 2(a–c) shows the XRD patterns of the different perovskite films. The PbI_2_ peaks at 12.7° indicate the incomplete conversion from PbI_2_ to MAPbI_3_, which causes variations in the stoichiometry of the resultant films. It was reported that the penetration depth of CH_3_NH_3_I into a compact PbI_2_ layer is limited to a few tens of nanometer from the surface^24–26^. For the low deposition rate of R_1_, a distinct peak for PbI_2_ can be seen in Fig. 2(a), which is ascribed to the inhibition of MAI diffusion caused by the formation of a compact MAPbI_3_ perovskite film on the PbI_2_ surface. At the rate of R_2_, the fabricated film shows a strong perovskite peak with the weakest peak for PbI_2_ among the three samples. It is assumed that the pores on the surface of the PbI_2_ film facilitate the permeation of MAI, which is beneficial for the conversion of PbI_2_ to perovskite. Figure 2(e) shows that a uniform perovskite film without pinholes is formed across the entire SEM image of the top surface. In contrast, the R_3_-based film shows an obvious residual PbI_2_ XRD peak that is even stronger than the perovskite peak, as illustrated in Fig. 2(c). Thus, a high-quality perovskite film could be fabricated by controlling the deposition rate of PbI_2_ at R_2_.

**Figure 3:**
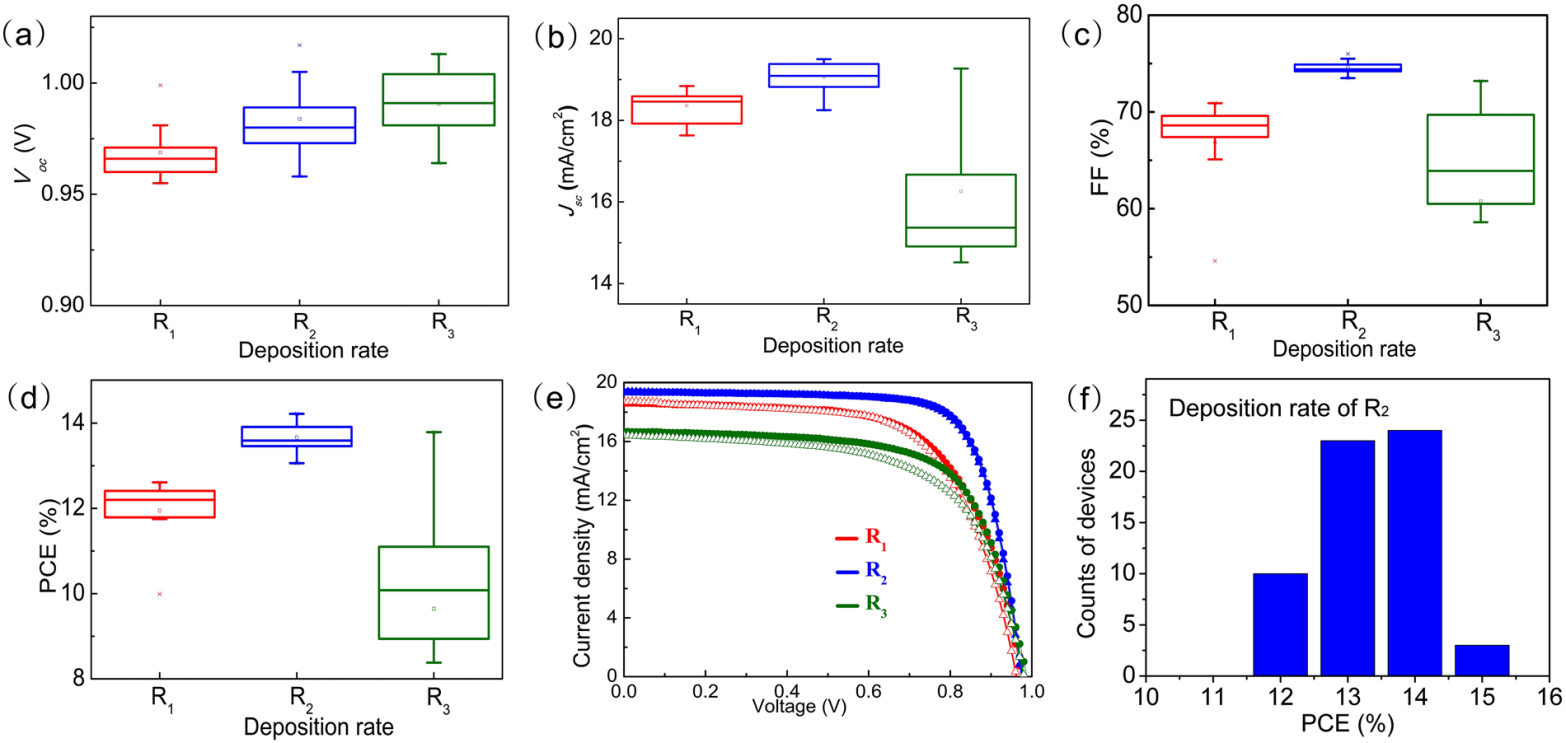
(**a**) Statistical distributions of *V*_*oc*_, *J*_*sc*_, FF and the corresponding PCE of the different groups of perovskite solar cells; the device photovoltaic parameters were determined from reverse scanning. Each group consists of 15 cells. (**e**) *J*-*V* curves of the devices fabricated from the films prepared at different PbI_2_ deposition rates. (**f**) Device performance distribution for 60 devices in three batches.

As the key material in perovskite solar cells, the perovskite films fabricated with different PbI_2_ deposition rates also have an important influence on the final photovoltaic performance. We first verified the principle of fabricating planar PSCs on a rigid substrate with the structure glass/ITO/C_60_/perovskite/spiro-OMeTAD/MoO_3_/Au^27^. The details of the fabrication process are described in the methods section. Except for the different deposition rates of PbI_2_ films, the other conditions were kept the same for all cells. The photovoltaic performance of the PSCs based on different deposition rates of PbI_2_ were measured under AM 1.5 G (100 mW cm^−2^) light illumination with a metal mask of 0.16 cm^2^, and illustrated in Fig. 3(a–d). The evolution of the device performance is consistent with the trends in the film and crystal quality observed from the SEM and XRD data. Compared with the devices fabricated at R_1_, all the photovoltaic parameters increased for the devices based on the R_2_ film due to the improved conversion to perovskite. This result is consistent with research from other groups, where the optimized performance was obtained for a higher PbI_2_ deposition rate^28^. However, when the deposition rate increased to 40 Å s^−1^, the device PCE decreased due to the presence of more unreacted PbI_2_ in the perovskite film. In contrast, the samples fabricated from the R_1_- and R_3_-based films exhibit smaller *J*_*sc*_ values and lower FFs, which is caused by the less complete transfer reaction from PbI_2_ to perovskite. The presence of a small amount of residual PbI_2_ is observed in many of the high-efficiency devices reported in the literature. Beneficial effects such as grain boundary passivation and hole-blocking effects have been proposed^29,30^. The *J*-*V* characteristics deliver a short-circuit current density (*J*_*sc*_) of 19.8 mA cm^−2^, an open circuit voltage (*V*_*oc*_) of 1.00 V, a fill factor (FF) of 0.76 and an efficiency of 15.04% for the device fabricated from the film prepared at a 20 Å s^−1^ deposition rate of PbI_2_. With the high-quality perovskite thin film, small-area devices using the optimized method show an average PCE of 13.7% ± 0.7%. It has been reported that planar perovskite solar cells are more susceptible to the hysteresis effect^31,32^. Thus, the perovskite solar cells based on the R_2_ deposition rate were measured under both reverse and forward scans and exhibited an almost negative hysteresis.

**Figure 4:**
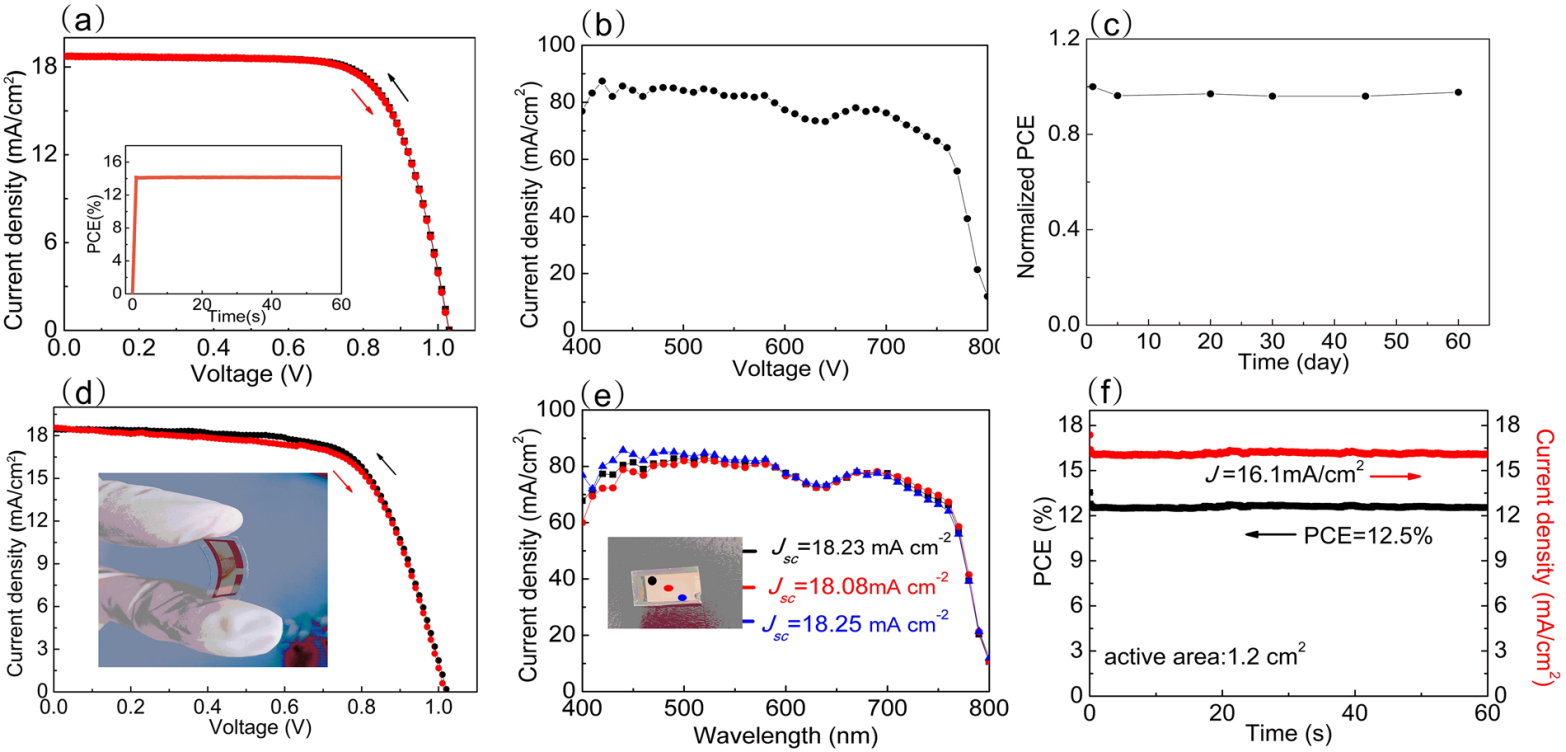
(**a**) *J-V* curve of the flexible perovskite solar cell with a metal mask of 0.16 cm^2^; the inset is the steady-state PCE at a bias of 0.81 V. (**b**) The corresponding EQE curve. (**c**) Long-term stability of the device with an area of 0.16 cm^2^ stored in a glovebox. (**d**) *J-V* curve of the flexible perovskite solar cell (calculated area: 1.2 cm^2^) without the metal mask. (**e**) EQE curves of the device at three selected spots. (**f**) The steady-state photocurrent density and PCE of the large-area perovskite solar cells (1.2 cm^2^) at a bias of 0.78 V.

Considering that the materials and techniques used above are all processed at low temperature (not higher than 150 °C), we directly applied these techniques to an ITO-PEN substrate without any modification. The photovoltaic characteristics of the flexible MAPbI_3_ devices with the above-optimized fabrication parameters and the same measurement conditions are shown in Fig. 4(a) and summarized in Table 1. The small-area flexible device shows PCEs of 13.9% under the reverse scan (*V*_*oc*_ to *J*_*sc*_) and 13.8% under the forward scan (*J*_*sc*_ to *V*_*oc*_). The inset of Fig. 4(a) shows a corresponding steady-state efficiency of 14.0% at a bias close to the initial maximum power point of 0.81 V. During a 60 second illumination period, the unencapsulated device shows a stable photocurrent density and PCE as a function of time. Figure 4(b) shows a typical external quantum efficiency curve (EQE) of the device. The integrated short-circuit current density is 18.5 mA cm^−2^ under the AM 1.5 G spectrum, which matches well with the *J*-*V* results. Upscaling the fabrication process, large area flexible device with an active area of 1.2 cm^2^ can be obtained as in Fig. 4(d). It shows a *V*_*oc*_ of 1.02 V, a *J*_*sc*_ of 18.4 mA cm^−2^, an FF of 0.68 and thus a PCE of 12.8% under the reverse scan. And under the forward scan, the device shows a *V*_*oc*_ of 1.02 V, a *J*_*sc*_ of 18.6 mA cm^−2^, an FF of 0.66 and thus a PCE of 12.5%. The EQE spectra at three spots located at the center and two corners of the device were measured and show small variations in the *J*_*sc*_, as shown in Fig. 4(e), which provides evidence for the uniformity of the perovskite film over a large area. Compared with the small-area device, an increase in the active area from 0.16 cm^2^ to 1.2 cm^2^ resulted in a 4% lower FF, which we ascribe to the series resistance of the ITO. In addition, long term stability test of the flexible perovskite solar cells was performed and the cells showed promising result with only 10% PCE decrease over 1000 h in Fig. 4(c). Besides highly electron selectivity and low temperature process, C_60_ as ETL has been proven with good stability in perovskite solar cells^17,33^.

**Table 1:** Photovoltaic performance parameters extracted from the *J*-*V* measurements under standard AM 1.5 G illumination (100 mW cm^−2^) of the device. The data were determined from 10 devices. The series resistance was calculated from the best device.

Cell type	Active area (cm^2^)	Scan direction	V_oc_ (V)	J_sc_ (mA cm^−2^)	FF	PCE (%)	Best PCE (%)	R_s_ (Ω)
F-PSC	0.16	Reverse	1.01 ± 0.02	18.1 ± 0.6	0.70 ± 0.04	12.6 ± 1.3	13.9	7
		Forward	1.00 ± 0.03	18.1 ± 0.7	0.69 ± 0.05	12.5 ± 1.3	13.8	
F-PSC	1.2	Reverse	1.01 ± 0.01	17.6 ± 0.6	0.60 ± 0.05	11.6 ± 1.1	12.8	10
		Forward	1.00 ± 0.02	17.0 ± 0.5	0.61 ± 0.05	11.1 ± 1.3	12.5	
F-Module	16	Reverse	4.90 ± 0.15	3.3 ± 0.3	0.50 ± 0.05	7.8 ± 0.5	8.6	76
		Forward	4.90 ± 0.10	3.3 ± 0.3	0.48 ± 0.02	7.3 ± 0.6	8.3	

**Figure 5:**
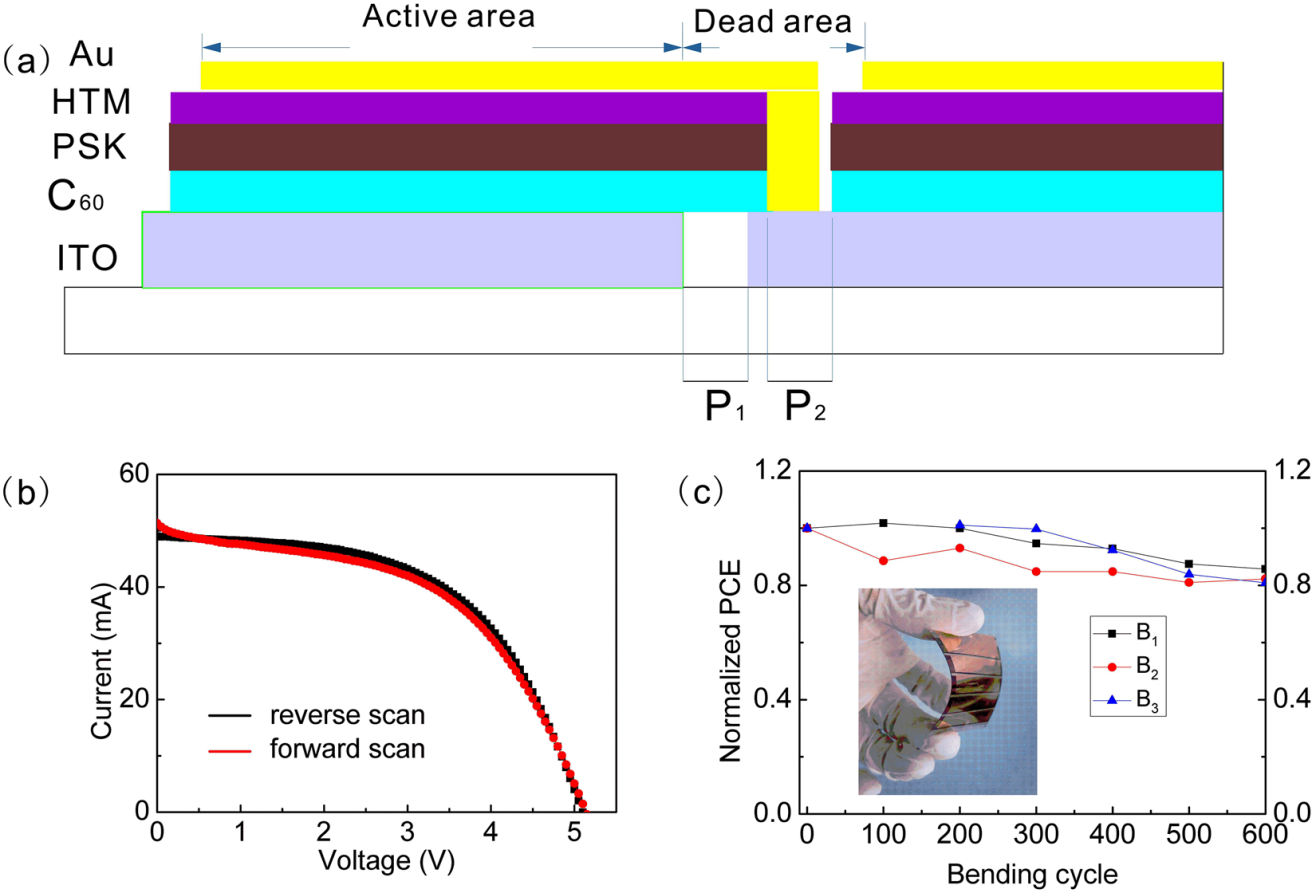
(**a**) Scheme showing the interconnection of the sub-cells in the perovskite thin film solar module with the patterning of the ITO bottom contact (P_1_) and the series connection of adjacent cells (P_2_). (**b**) *I-V* curve of the perovskite solar module with a calculated area of 16 cm^2^ tested under 100 mW cm^−2^ illumination. (**c**) Normalized performance of the flexible perovskite solar module versus the number of bending cycles; bending the module parallel to the etching line (B_1_), perpendicular to the etching line (B_2_), and in both of the above two directions (B_3_).

From the results shown above, we can confirm that pinhole-free and uniform perovskite layers with good electrical properties were obtained using the sequential evaporation/spin-coating process, and therefore, flexible perovskite solar modules were fabricated. The module of the flexible perovskite solar cell had a large size of 5 cm × 5 cm, which was composed of 5 serially connected sub-cells with a 3.2 cm^2^ aperture area for each. In a two-step patterning process with a femtosecond laser, the sub-cells were combined in a standard serial interconnection geometry, as shown in Fig. 5(a). After coating the module with hole transport material (HTM), a femtosecond laser was used to cut the ETL/perovskite/HTM layer, and then the electrode was evaporated to connect each sub-cell. Figure 5(b) shows the *I*-*V* curve of the flexible perovskite solar module. When measured under the reverse scan, the module exhibits a *V*_*oc*_ of 5.10 V (equivalent *V*_*oc*_ 1.02 V), a *I*_*sc*_ of 49.1 mA (equivalent *J*_*sc*_ 15.3 mA), an FF of 0.55 and thus a PCE of 8.6%. When measured under the forward scan, the module exhibits a *V*_*oc*_ of 5.14 V (equivalent *V*_*oc*_ 1.03 V), a *I*_*sc*_ of 51.2 mA (equivalent *J*_*sc*_ 16.0 mA), and an FF of 0.50, resulting in a PCE of 8.2%. Compared with a typical 1.2-cm^2^-area device, the module shows decreased *J*_*sc*_ and FF values, which may be due to the interconnected width of the series resistance (a series resistance of 76 Ω) contributed by ITO. To verify the efficiency from the *I*-*V* curves, the steady-state output at a bias of 2.85 V was measured. The flexible module shows a nearly constant PCE of 7.6% during the measurement under the 100 mW cm^−2^ illumination shown in Supplementary Fig. S3.

A circular bending test was carried out on the flexible perovskite solar module to check the reliability. The module was tested with a radius of curvature of 32 mm in three different bending tests: bending parallel to the etching line, perpendicular to the etching line and both parallel and perpendicular to the etching line. The flexible devices exhibit decreased photovoltaic performance with bending the module, as shown in Fig. 5(c). The significant change in the flexible module performance upon multiple bending cycles originates from the decreased FF, which drops to 80% of its initial performance after 600 cycles in Supplementary Fig. S4. Most of the layers in the device, except for the ITO film, had high flexibility^34^. Specifically, the resistance of the ITO-PEN substrate was tested as a function of the number of bending cycles. The results clearly show a trend of increasing resistance with increasing number of bending cycles, as shown in Supplementary Table S2. This increasing resistance of ITO is in consistent with the decreased FF of the module.

## Discussion

In conclusion, we have demonstrated a promising sequential deposition method for preparing CH_3_NH_3_PbI_3_ films at an ultrafast PbI_2_ evaporation rate of 20 Å s^−1^, through which we obtained uniform and pinhole-free perovskite films in a short time. The rate-controlled PbI_2_ deposition was investigated in detail, and devices were fabricated on flexible substrates. A 16 cm^2^ flexible perovskite solar module based on the MAPbI_3_ film deposited from an optimized PbI_2_ film was fabricated and had an efficiency of more than 8%. This strategy shows great potential for paving the way to the manufacture of large perovskite solar modules.

## Methods

### Materials

Unless specified otherwise, all chemicals were purchased from either Alfa Aesar or Sigma-Aldrich and used as received. 2,2′,7,7′-Tetrakis-(N,N-di-4-methoxyphenylamino)-9,9′-spirobi-fluorenes (spiro-OMeTAD) was purchased from Shenzhen Feiming Science and Technology Co., Ltd., and MAI was purchased from Xi’an Polymer Light Technology Co., Ltd.

### Preparation of devices

Indium tin oxide coated glass (ITO-Glass) and polyethylene naphthalate (ITO-PEN) were patterned by a femtosecond laser, followed by ultrasonic cleaning in detergent water, deionized water and ethanol for 15 min each. The substrates were then dried under an N_2_ flow and treated with plasma for 5 min to remove any organic residues. Fullerene C_60_ was thermally evaporated onto the ITO-coated substrates with a thickness of 10 nm under a deposition pressure of 8 × 10^–6^ mbar at a deposition rate of 0.1 Å s^−1^. The 160 nm PbI_2_ films were then thermally evaporated on the C_60_ underlayer with controlled deposition rates within the range between 0.5 Å s^−1^ and 40 Å s^−1^, as monitored by a quartz crystal microbalance sensor. After the PbI_2_ deposition process, the samples were transferred to a spin coater in an N_2_-filled glovebox for the subsequent chemical conversion steps. A 56 mg ml^−1^ methyl-ammonium iodide (MAI) solution in ethanol containing 24 μl of 2-methoxyethanol was spread on the whole PbI_2_ film and then spin-coated at a rate of 3000 rpm for 30 s. The yellow film turned brown during the spin-coating and was then annealed at 150 °C on a hotplate outside of the glovebox for 10 min. To prepare the HTM layer, a solution of spiro-OMeTAD dissolved in ethyl acetate^35^ (41.6 mg ml^−1^, 17 μl ml^−1^ 4-tert-butylpyridine, 7.8 μl ml^−1^ stock solution of 500 mg ml^−1^ lithium bis trifluoromethylsulfonyl imide in acetonitrile) was dropped onto the perovskite layer while spinning at a rotation rate of 2000 rpm. For the modules, P_1_ patterning of the module was performed with a femtosecond laser (Wuhan Hongtuo). After the HTM layer was deposited, the C_60_/perovskite/spiro-OMeTAD layers were fully removed to ensure low contact resistance at the interconnected space through P_2_ patterning. Finally, 10 nm MoO_3_ and 60 nm Au were successively evaporated through a properly designed mask under high vacuum (4 × 10^−6^ mbar) to complete the devices for tests.

### Characterization

The photocurrent density-voltage characteristics of the devices were measured with a scan rate of 0.01 V s^−1^ under standard simulated AM 1.5 G illumination (100 mW cm^−2^) using a solar simulator (Oriel 94023 A, 300 W), which was calibrated using a standard Si solar cell (Oriel, VLSI standards). The external quantum efficiency (EQE) curves were obtained using monochromatic incident light produced by a power source (Pharos Technology) with a monochromator. Data acquisition was accomplished under DC mode with a power meter. The morphologies and microstructures of the deposited PbI_2_ films and perovskite films were investigated using a field-emission scanning electron microscope (FE-SEM, Zeiss Ultra Plus) and an X-ray diffractometer (XRD, D8 Advance).

## Electronic supplementary material

Supplementary Information
