# Supplementary material for: An efficient, flexible perovskite solar module exceeding 8% prepared with an ultrafast PbI2 deposition rate
Source: Sci Rep. 2018 Jan 11;8:442. doi: 10.1038/s41598-017-18970-y (PMC5765045; doi:10.1038/s41598-017-18970-y)
Supplement: Supplementary file 1 — Supplementary Information [file 41598_2017_18970_MOESM1_ESM.doc]

**Supplementary Information**

**An efficient, flexible perovskite solar module exceeding 8% prepared with an ultrafast PbI_2_ deposition rate**

^1^Kunpeng Li, ^1^Junyan Xiao, ^1^Xinxin Yu, ^1^Tianhui Li, ^1^Da Xiao, ^1^Jiang He, ^1^Peng Zhou, ^1^Yangwen Zhang, ^2^Wangnan Li, ^1^Zhiliang Ku, ^1^Jie Zhong, ^1^Fuzhi Huang, ^1^Yong Peng* and ^1,3^Yibing Cheng

1 State key lab of advanced technology for materials synthesis and processing, Wuhan University of Technology, China, 430070

2 Hubei key laboratory of low dimensional optoelectronic materials and devices, Hubei University of Arts and Science, Xiangyang China 441053

3 Department of materials science and engineering, Monash University, Australia, VIC 3800


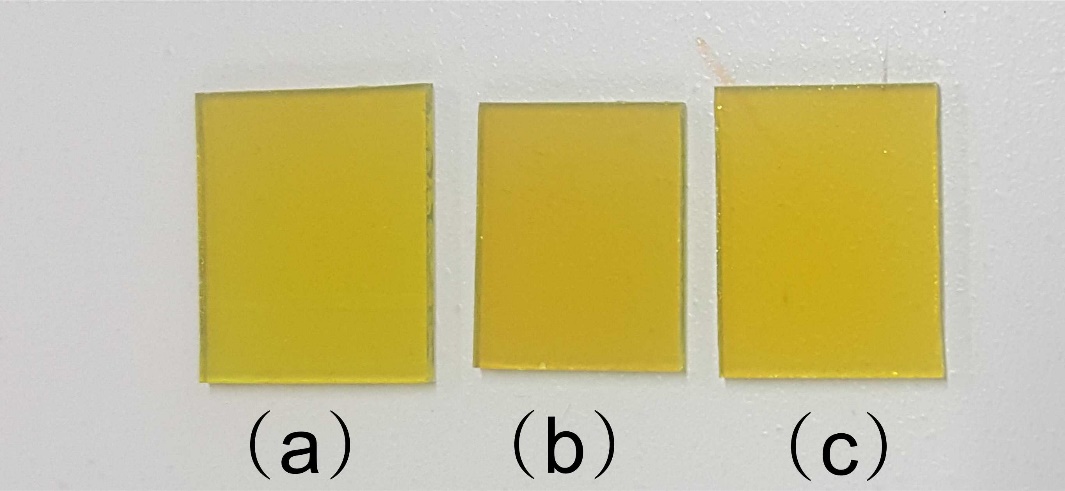


**Figure S1.** Photograph of the different rates deposited PbI_2_ films. The deposition rate of PbI_2_: (a) 0.5 Å s^-1^ (b) 20 Å s^-1^ (c) 40 Å s^-1^

**Table S1** Results of (001) XRD peaks from PbI_2_ films deposited at different rates.

| Deposition rate | 2 theta  (degree) | FWHM (degree) | Crystalline grain size |
| --- | --- | --- | --- |
| 0.5 | 12.73 | 0.09348 | 84 nm |
| 20 | 12.73 | 0.1122 | 70 nm |
| 40 | 12.75 | 0.1309 | 60 nm |

Note: the crystalline grain size was calculated from the Scherrer equation equitation D=0.89λ/βCosθ, where D is the crystalline grain size, λ is the X- ray wavelength, β is (FWHM), θ is the Bragg diffraction angle of XRD peak (degree).


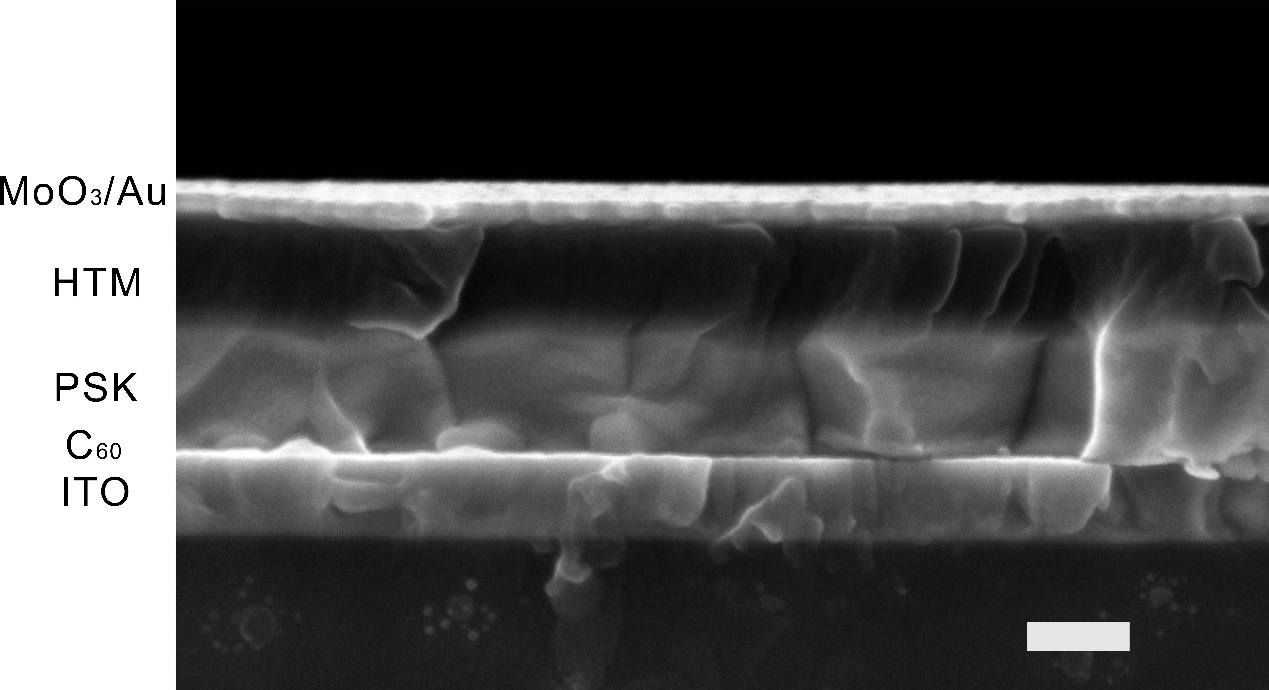


**Figure S2.** Cross sectional SEM image of the device with the structure of ITO/C_60_/perovskite/HTM/MoO_3_/Au, the hole transport layer material (HTM) layer is the 2,2’,7,7’-Tetrakis-(N, N-di-4-methoxyphenylamino)-9,9’-spirobi-fluorenes (spiro-OMeTAD). The scale bar indicates 200 nm.


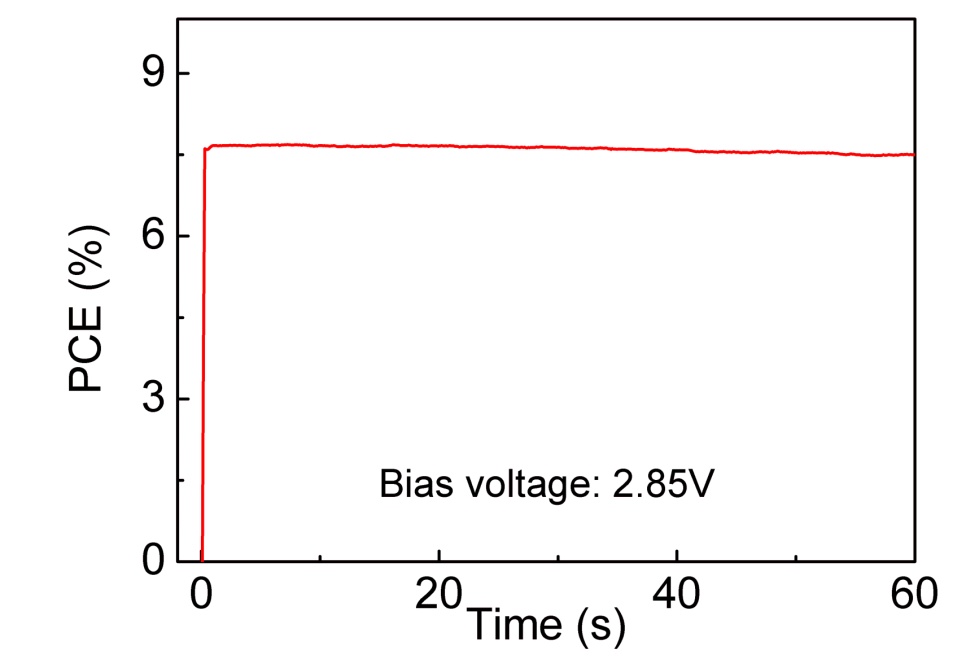


**Figure S3.** The steady state PCE output which was taken at a voltage bias of 2.85V as a function of time.

**Table S2** Resistance versus bending cycles of the flexible ITO/PEN substrates.

| Bend cycle | 0 | 100 | 200 | 300 | 400 | 600 |
| --- | --- | --- | --- | --- | --- | --- |
| Resistance (Ω) | 53 | 59 | 64 | 68 | 72 | 80 |

Note: the resistance between two selected spots on the ITO-coated PEN substrates was measured as a function of the bending cycles with a radius of curvature of 32 mm.


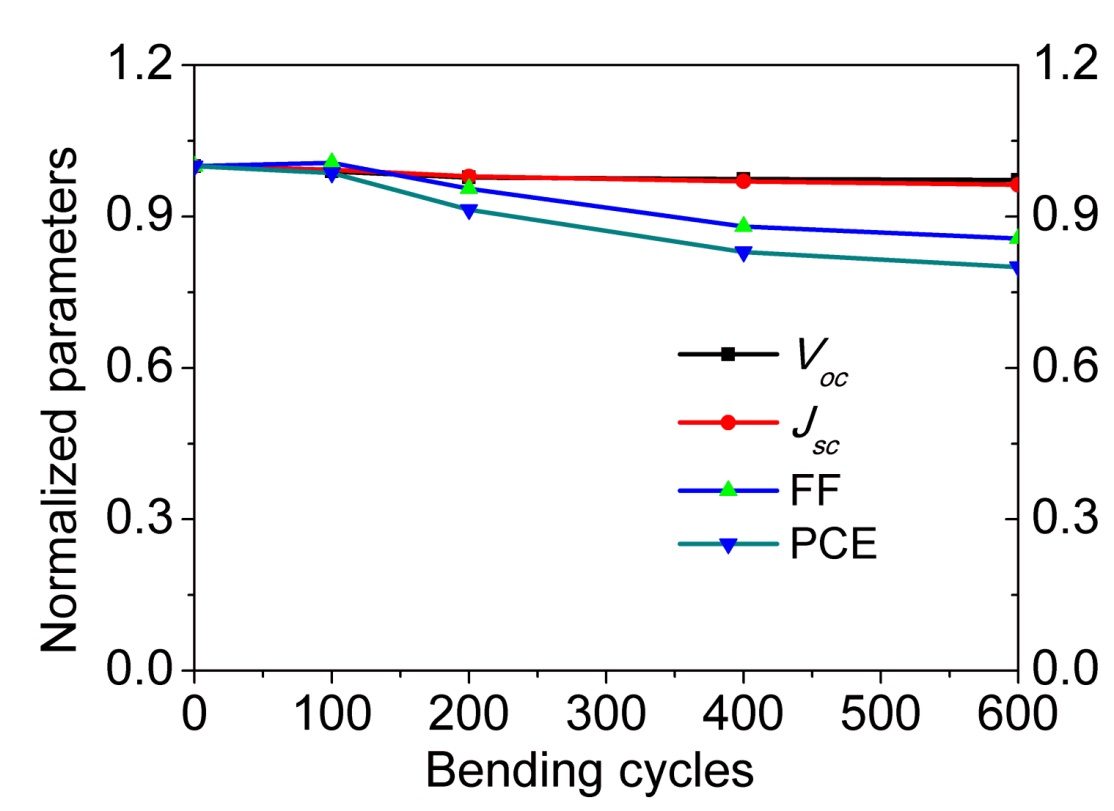


**Figure S4**. Normalized performance parameters of a flexible perovskite solar module versus bending cycles.
